# Pneumococci in biofilms are non-invasive: implications on nasopharyngeal colonization

**DOI:** 10.3389/fcimb.2014.00163

**Published:** 2014-11-06

**Authors:** Ryan P. Gilley, Carlos J. Orihuela

**Affiliations:** Department of Microbiology and Immunology, Center for Airway Inflammation Research, The University of Texas Health Science Center at San AntonioSan Antonio, TX, USA

**Keywords:** *Streptococcus pneumoniae*, biofilms, colonization, virulence, transmission

## Abstract

*Streptococcus pneumoniae* (the pneumococcus) is an opportunistic pathogen that colonizes the human nasopharynx asymptomatically. Invasive pneumococcal disease develops following bacterial aspiration into the lungs. Pneumococci within the nasopharynx exist as biofilms, a growth phenotype characterized by surface attachment, encasement within an extracellular matrix, and antimicrobial resistance. Experimental evidence indicates that biofilm pneumococci are attenuated vs. their planktonic counterpart. Biofilm pneumococci failed to cause invasive disease in experimentally challenged mice and *in vitro* were shown to be non-invasive despite being hyper-adhesive. This attenuated phenotype corresponds with observations that biofilm pneumococci elicit significantly less cytokine and chemokine production from host cells than their planktonic counterparts. Microarray and proteomic studies show that pneumococci within biofilms have decreased metabolism, less capsular polysaccharide, and reduced production of the pore-forming toxin pneumolysin. Biofilm pneumococci are predominately in the transparent phenotype, which has elevated cell wall phosphorylcholine, an adhesin subject to C-reactive protein mediated opsonization. Herein, we review these changes in virulence, interpret their impact on colonization and transmission, and discuss the notion that non-invasive biofilms are principal lifestyle of *S. pneumoniae*.

## Introduction

*Streptococcus pneumoniae* (the pneumococcus) is a leading cause of community-acquired pneumonia (CAP), sepsis, and meningitis throughout the world despite the existence of multiple effective vaccines (Bennett et al., 2014). This Gram-positive, encapsulated bacterium asymptomatically colonizes the human nasopharynx where carriage can last for months (Gray et al., 1980). In susceptible individuals, usually the very young and elderly, aspiration of pneumococci can lead to pneumonia and subsequently invasive pneumococcal disease (IPD). At any given time approximately 40% of children and 15% of adults are colonized (Crook et al., 2004; Huang et al., 2009). Annual global IPD burden is roughly 14.5 million cases resulting in 800,000 deaths in children under the age of 5 and a case fatality rate surpassing 20% in the elderly (O'Brien et al., 2009; Heron, 2012; Naucler et al., 2013).

**Figure 1:**
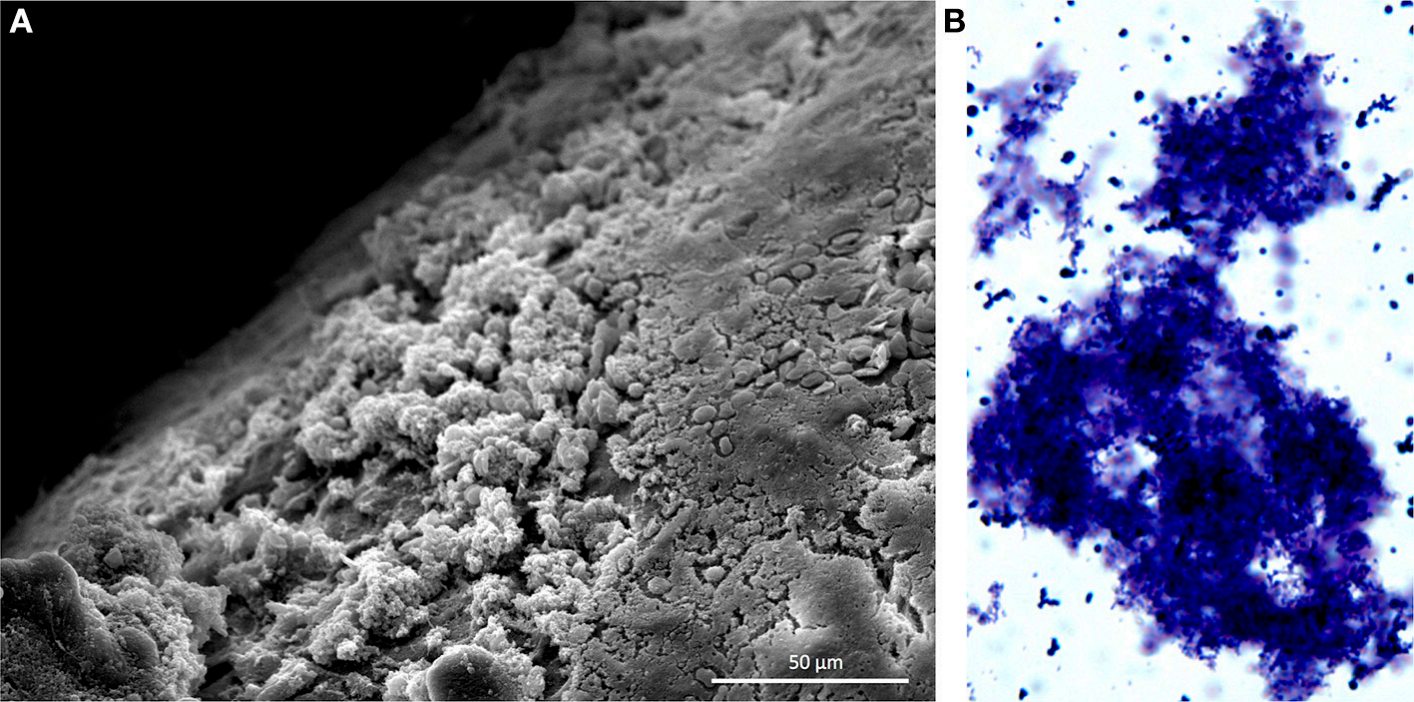
**Pneumococcal biofilms form in the nasopharynx. (A)** Scanning electron microscopy image of *S. pneumoniae* biofilms formed on the nasal septum of a mouse. Mice were experimentally colonized 7 days prior. Biofilms are the non-contiguous aggregates on the left. (**B)** *S. pneumoniae* biofilm aggregate in nasopharyngeal lavage fluid. Sample was collected from mouse 14 days after experimental colonization. Pneumococci were stained with crystal violet and visualized with a light microscope at 400X. Image credit: Krystle Blanchette.

*S. pneumoniae* in sputum and blood samples from individuals with IPD are primarily in the form of lancet-shaped diplococci; the same morphology observed when grown planktonically in media. Growth as diplococci or short chains is now recognized to help *S. pneumoniae* evade stochastic alternative pathway mediated complement deposition and opsonophagocytosis (Dalia and Weiser, 2011). Within the past 15 years it has become evident that the pneumococcus also forms biofilms *in vivo* during nasopharyngeal colonization (Figure 1) and otitis media (Hoa et al., 2009; Reid et al., 2009). Biofilms are aggregates of surfaced attached bacteria encased within an extracellular matrix (ECM). The ECM, which *in vivo* is composed of host factors, polysaccharides, and extracellular DNA, is now understood to protect bacteria from the host immune system and desiccation (Moscoso et al., 2006); the latter being important during pneumococcal fomite transmission (Walsh and Camilli, 2011). Importantly, biofilm pneumococci have been shown to be decisively less virulent than their planktonic counterparts. This review focuses on how *S. pneumoniae* modulates its virulence during biofilm formation and why this may promote long-term, asymptomatic colonization. We also discuss the increasingly evident role biofilms play during pneumococcal transmission on fomites.

## Biofilm pneumococci are avirulent

Given the importance of biofilms in recalcitrant infections and for *S. pneumoniae* in the middle ear during otitis media (Reid et al., 2009; Chauhan et al., 2014), initial studies examining pneumococcal biofilms sought to associate the ability to form biofilms with enhanced virulence (Munoz-Elias et al., 2008; Lizcano et al., 2010). However, the ability to form biofilms *in vitro* could not be linked to the anatomical site from which a clinical isolate was obtained (i.e., nasopharynx of an asymptomatic carrier or blood from individual with IPD), nor the ability of the isolate to cause bacteremia in an infectious mouse model (Hall-Stoodley et al., 2008; Lizcano et al., 2010). Importantly, these and other studies have shown that *in vitro* biofilm formation was most enhanced for mutants that lacked capsular polysaccharide (CPS) (Moscoso et al., 2006). CPS mutants are avirulent due to their inability to prevent opsonophagocytosis (Hyams et al., 2010). Thus, the fact that unencapsulated mutants form more robust biofilms suggested a direct disconnect between pneumococcal biofilm formation and its propensity for invasive disease.

**Figure 2:**
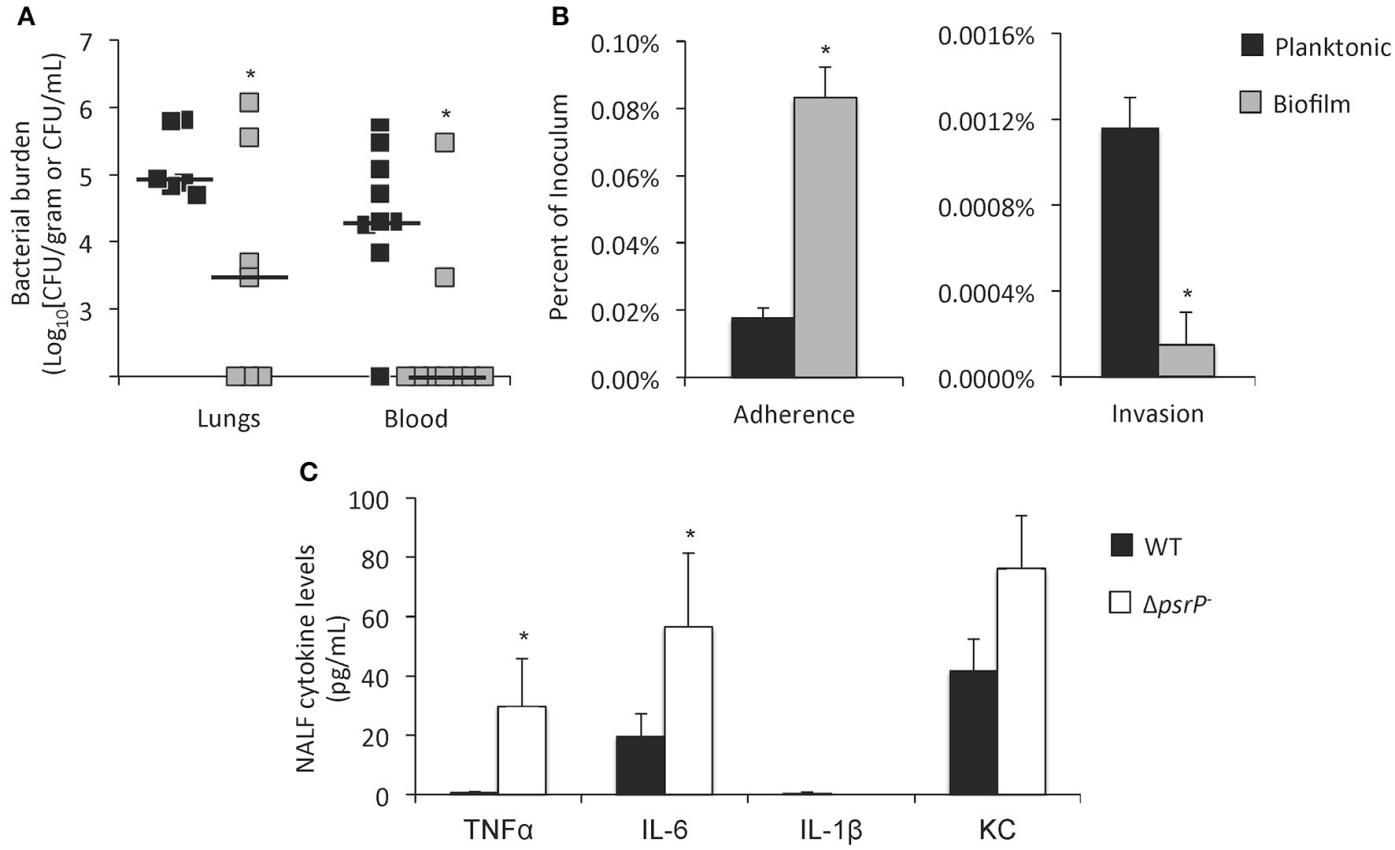
**Biofilm pneumococci are less virulent and elicit a weaker immune response than their planktonic counterparts**. **(A)** Bacterial titers in the lungs and blood of BALB/c mice challenged intratracheally with 10^5^ CFU of planktonic or biofilm derived *S. pneumoniae* (each square = individual mouse; *n* = 6–8). From: Sanchez et al. (2011b). **(B)** Percentage of planktonic and biofilm derived *S. pneumoniae* that attached and invaded Detroit-562 pharyngeal cells *in vitro*. Percentages were calculated from the total inoculum. **(C)** Cytokine levels in nasopharyngeal lavage fluid (NALF) of colonized mice. Mice were challenged with wild type (WT) and PsrP-deficient (Δ*psrP*^−^) *S. pneumoniae* and NALF collected 7 days later. Note that Δ*psrP*^−^ does not form biofilms during colonization (*n* = 5). Panels **(B,C)** from: Blanchette-Cain et al. (2013). Asterisks denote a statistically significant difference (*P* < 0.05).

To directly test if pneumococci within biofilms were virulent, Sanchez et al. intratracheally challenged mice with equal colony forming units (CFU) of a virulent serotype 4 isolate grown to exponential (mid-logarithmic) phase in media or as a 3-day biofilm in a continuous flow-through reactor. They observed that only mice infected with planktonic pneumococci progressed to bacteremia while most of those challenged with biofilm pneumococci had negative blood cultures (Figure 2A) (Sanchez et al., 2011b). Studies by Blanchette-Cain et al. showed that pneumococci grown as a biofilm were hyper-adhesive yet uninvasive when tested *in vitro* on Detroit-562 pharyngeal epithelial cells (Figure 2B) (Blanchette-Cain et al., 2013). Marks et al. had similar results and showed that pneumococci grown as biofilms on fixed and live NCI-H292 bronchial epithelial cells neither invaded nor were internalized. Of note, Marks et al. showed that pneumococci recently dispersed from a biofilm due to an inflammatory signal, such as viral infection, were hyper-virulent with substantially greater capacity to cause invasive disease in mice than either biofilm pneumococci or pneumococci grown for a sustained period planktonically (Marks et al., 2013). Why recently dispersed pneumococci are more virulent than their sustained planktonic counterparts is not immediately clear, albeit two possibilities are that these bacteria carry biofilm ECM components that enhance their adhesive capacity, and major changes in gene expression profiles (Pettigrew et al., 2014). This observation helps to explain why viral infection is an established risk factor for the development of pneumococcal pneumonia (Brundage, 2006; McCullers, 2006).

## Reduced capsular polysaccharide during biofilm growth

CPS is the principal virulence determinant for *S. pneumoniae* and exists in >90 identified serotypes (Bennett et al., 2014). In addition to resisting opsonophagocytosis (Melin et al., 2010), the negative or neutral charge of CPS plays an important role in helping the pneumococcus evade entrapment in mucus (Nelson et al., 2007). The necessity of CPS for IPD is exhibited by the fact that all invasive strains of *S. pneumoniae* are encapsulated whereas unencapsulated pneumococci are infrequent and usually only associated with topical eye infection (Barker et al., 1999).

Multiple investigators have reported an inhibitory role for CPS during *in vitro* biofilm formation with capsule deficient mutants forming substantially more robust biofilms than their encapsulated parent strain (Moscoso et al., 2006; Qin et al., 2013). Allergucci and Sauer showed that biofilms formed by a serotype 3 isolate were in large part composed of spontaneous mutants deficient in CPS related genes (Allegrucci and Sauer, 2007). Marks et al. have added evidence that this may occur *in vivo* by showing that unencapsulated pneumococci form more robust biofilms on the surface of epithelial cell monolayers (Marks et al., 2012). In fact, the presence of a capsule was shown to inhibit unencapsulated pneumococci from forming robust biofilms in mixed *in vitro* cultures (Domenech et al., 2009). Yet, CPS production is required for efficient *in vivo* colonization (Shainheit et al., 2014), indicating that during colonization the pneumococcus must strike a balance between CPS hindrance of biofilm formation and resistance to host defense.

Gene expression analyses using qRT-PCR and microarrays have shown that genes within the CPS operon were downregulated during *in vitro* biofilm formation vs. planktonic growth (Oggioni et al., 2006; Sanchez et al., 2011b). Moreover, the amount of capsule detected and the enzymes responsible for CPS production were substantially lower for biofilm vs. planktonic grown pneumococci as detected by ELISA and MALDI-TOF (Sanchez et al., 2011a,b). In agreement with dynamic changes in CPS production, pneumococci reduce capsule thickness once in contact with epithelial cells (Hammerschmidt et al., 2005). This is supported by microarray gene analysis of cells in contact with respiratory epithelial cells *in vitro* (Orihuela et al., 2004b). Thus, biofilm pneumococci reduce levels of CPS making them more susceptible to phagocytosis following aspiration.

## Phase variation

*S. pneumoniae* oscillates between an opaque phase variant that produces high levels of CPS and low levels of cell wall teichoic acid, and a transparent phase variant with low CPS and high cell wall teichoic acid (Weiser et al., 1994). The basis for phase variation is now understood to be epigenetic, with alternate methylation patterns on genes (Manso et al., 2014). Due to negative selection for the transparent phase by phagocytes, opaque variants predominate in the blood (Kim and Weiser, 1998). In contrast, the transparent phenotype is better able to adhere to cells and thus predominates in the nasopharynx (Weiser et al., 1994). Of note, Sanchez et al. have shown that *in vitro* biofilms are primarily composed of the transparent variant, despite the seed cultures used to initiate the biofilm being mostly opaque (Sanchez et al., 2011b).

In its transition from opaque to transparent, the pneumococcus loses virulence potential while enhancing its ability to adhere to host cells. As discussed, loss or a reduction in CPS enhances susceptibility to opsonophagocytosis yet is required for the exposure of surface adhesins that mediate bacterial attachment to host cells (Ring et al., 1998). Critically, cell invasion occurs for planktonic but not biofilm pneumococci (Blanchette-Cain et al., 2013). The increased amount of teichoic acid carried by the transparent variant also makes it subject to recognition by C-reactive protein (CRP), resulting in activation of complement (Kim et al., 1999). However, phosphorylcholine residues present on teichoic acid allow the pneumococcus to bind to the host ligand platelet-activating factor (PAFr) receptor on host cells (Cundell et al., 1995).

Despite loss of capsule and increased exposure of teichoic acid, pneumococci in biofilms are resistant to opsonophagocytosis (Yuste et al., 2007). One reason for this includes that CRP binding to phosphorylcholine is competed with by members of the choline-binding protein family (Mukerji et al., 2012), such as the adhesin Choline-binding protein A (CbpA) which is upregulated during transparent phase growth as well as in biofilms (Sanchez et al., 2011b). CbpA is also a key inhibitor of complement deposition through its binding to Factor H and complement component C3 (Cheng et al., 2000; Dave et al., 2001). Another choline-binding protein that plays a key role in complement inhibition includes Pneumococcal surface protein A (PspA), which prevents classical complement activation in a C1q dependent manner (Tu et al., 1999; Yuste et al., 2007; Mukerji et al., 2012). Importantly, the opaque variant has been suggested to play a critical role in the formation of the ECM (Trappetti et al., 2011). Of note, gene expression studies for biofilms and transparent pneumococci do not entirely overlap. Thus, phase variation is an important aspect of pneumococcal biofilm formation but is not entirely responsible for its phenotype.

## Downregulation of metabolic processes and modulated virulence gene expression

Antimicrobial resistance is one of the defining properties of biofilms and has been extensively documented for biofilm pneumococci, particularly in the context of recurring otitis media (Stewart and Costerton, 2001; Hall-Stoodley et al., 2008). Why, the ability of an isolate to form well-structured biofilms *in vivo* was correlated with resistance to high concentrations of gentamycin (Marks et al., 2012). Enhanced resistance to antimicrobials in biofilm pneumococci may be due to a decrease in metabolic rate, which also confers resistance to antimicrobials targeting cell wall, protein synthesis, and DNA replication. The ECM also serves as an inhibitor or off-target for antimicrobials. This topic is extensively reviewed elsewhere (Domenech et al., 2012). Once aspirated, a reduced metabolic rate would presumably impair the ability of biofilm pneumococci to respond in a timely fashion to hostile host factors present in the lower respiratory tract.

Along such lines, planktonic and biofilm *S. pneumoniae* are now recognized to have distinct protein and gene expression profiles. Using qRT-PCR, Oggioni et al. showed that the gene expression profile of virulence-associated genes of different strains isolated from the blood were more similar to that of planktonic growth in broth, whereas the same strain isolated from the lungs, brain, or nasopharynx of infected mice was more similar to that of *in vitro* biofilms (Oggioni et al., 2006). Microarray analysis of *in vitro* grown planktonic vs. biofilm pneumococci showed that biofilm pneumococci downregulated genes involved in protein synthesis, energy production, metabolism, CPS production; along with the virulence genes that encode the pneumococcal pilus, which has been shown to be an invasin (Barocchi et al., 2006), and the pore-forming toxin pneumolysin (Sanchez et al., 2011b). Pneumolysin has been demonstrated to be required for systemic bacteremia and host cell damage and inflammation (Orihuela et al., 2004a; Mitchell and Dalziel, 2014), thus its down regulation would most likely compromise virulence. Yet pneumolysin has also been shown to contribute toward *in vitro* biofilm formation (Shak et al., 2013). Thus, and like that for CPS, pneumolysin production is most likely fine-tuned to strike a balance with the host during colonization. In contrast, the genes encoding the adhesins PsrP, PavA as well as the previously discussed CbpA, were detected as being upregulated during biofilm growth (Sanchez et al., 2011b; Qin et al., 2013). These proteins may play a role in intra-species aggregation such as observed during *in vivo* biofilms, either by binding to other pneumococci directly or through bridging molecules such as fibronectin (Blanchette and Orihuela, 2012). The, why biofilm pneumococci do not invade cells remains unclear.

Mass spectroscopy (MS) based identification of proteins isolated from biofilm and planktonic cell lysates confirm profound differences between these two physiological growth states (Allegrucci et al., 2006; Sanchez et al., 2011a). One important caveat to this approach is that pneumococcal biofilms are in part composed of dead pneumococci and proteomic studies don't distinguish between proteins from live bacteria or those dead bacteria that have accumulated within the biofilm. When alive, these dead bacteria may have had a substantially different proteome. Nonetheless, and in agreement with microarray studies, MS of biofilm and planktonic cell lysates by Sanchez et al. found that the frequency of peptides corresponding to enzymes involved in protein synthesis and processing, energy metabolism, CPS production, and proteins involved in transcription, regulation and DNA binding, as well as the virulence determinants enolase, pyruvate oxidase (produces hydrogen peroxide), and pneumolysin were less frequent in biofilm lysates than planktonic lysates (Sanchez et al., 2011a). The extent to which major differences occur in the proteome is further highlighted by the finding that antiserum from humans who recovered from IPD robustly recognized proteins in planktonic cell lysates but not biofilm cell lysates when tested by Western blot (Sanchez et al., 2011a). This provides evidence that the *in vivo* antigen protein profiles for colonization vs. invasive disease are considerably different, and that the overall productions of factors that mediate a response to a host or subvert the host response are altered.

## Host response to biofilm pneumococci

Only recently have investigators begun to examine how the host responds to biofilm pneumococci. Studies by Blanchette-Cain et al. have shown that biofilm pneumococci elicit significantly less Interleukin (IL)-6 and IL-8 from Detroit-562 pharyngeal epithelial cells than planktonic cultures. Similarly, biofilm pneumococci elicited less IL-6, IL-1β, and TNFα, from J774A.1 macrophages. *In vivo*, mutant pneumococci lacking the biofilm determinant PsrP, and thus unable to form *in vivo* biofilms, elicited greater TNFα, IL-6, IL-1β, and KC production in the nasopharynx of 7-day colonized mice vs. its parent strain (Figure 2C) (Blanchette-Cain et al., 2013). This was credited to the reduced tissue invasiveness of biofilm pneumococci, but as indicated may also involve reduced production of the toxin pneumolysin. Importantly, pneumococci may also actively suppress the host response in a way that has not yet been determined. For example studies have shown that Group B Streptococcus interacts with Siglec-5 and this dampens the host response (Carlin et al., 2009). Future studies examining this possibility are warranted.

Given the fact that the majority of individuals are colonized asymptomatically, we speculate that non-invasive pneumococci within biofilms promote long-term colonization and transmission through less vigorous activation of the innate immune response and therefore a delay in the onset of the adaptive response and their clearance. Yet, direct evidence for this is lacking with intranasal challenge of mice with PsrP-deficient or other mutants that are biofilm deficient not resulting in reduced bacterial titers in the nasopharynx when measured by CFU or qRT-PCR (Blanchette-Cain et al., 2013). Thus, studies are warranted to determine what the true physiological advantage of this immunoquiescent phenotype actually is and if it impacts the number of bacteria in the nasopharynx or long-term carriage.

## Biofilms as source of fomite transmission and conclusions

Multiple studies have shown that biofilm-derived pneumococci are more resistant to desiccation than their planktonic counterparts (Walsh and Camilli, 2011), with viable cells isolated from fomites over a period ten times longer than planktonic (Marks et al., 2014). Additionally, viable pneumococci have been recovered from a variety of desiccated surfaces in a day care setting: hands, books, and both hard and soft toys. Importantly, desiccated pneumococci recovered from fomites still retain colonization capabilities in a murine model, even with a normal inoculum (Walsh and Camilli, 2011; Marks et al., 2014). As is shown in Figure 1B, the pneumococcal aggregates can be sloughed from the nasopharynx and these aggregates most likely are a vehicle for transmission, possibly providing the bacteria with moisture and nutrients for an extended period outside the body. We point out that dispersed bacteria, as shown by Marks et al. (2013), are planktonic and may be a second vehicle for transmission following an inflammatory episode such as virus infection.

Most individuals carrying *S. pneumoniae* are colonized asymptomatically, thus the biofilm state is the major form by which the pneumococcus interacts with its host. Herein we have discussed how biofilm pneumococci are distinct from their planktonic counterparts. Specifically, pneumococci downregulate CPS, enhance expression of adhesins, shift toward the transparent phenotype, and lower the expression of metabolic processes and key virulence determinants that elicit a robust host response. Therefore, biofilm pneumococci seem to be exquisitely honed to the colonization phenotype at the expense of the invasive phenotype. There are many questions that remain to be answered; for example, direct evidence that the immunoquiescent phenotype confers a colonization advantage is lacking. This may be due to limitations in the current model systems and/or our ability to quantify bacteria *in vivo*. Additionally, does the pneumococcus rely on dispersal of biofilm aggregates or the spread of highly invasive biofilm dispersed planktonic pneumococci as the principle method for transmission, or are both effective? Perhaps different strains rely differently on these transmission methods. There are also infections that seem to be a mix of biofilms and planktonic bacteria, for example during otitis media. How these two physiological states impact the course of disease is unclear and warrants attention. In summary, a myriad of functional reasons can and do exist for why biofilm pneumococci are less virulent. A better understanding of the short-term survival and long-term evolutionary advantages would substantially enhance our understanding of pneumococcal biology, and may permit us to develop novel targets for bacterial clearance.

### Conflict of interest statement

The authors declare that the research was conducted in the absence of any commercial or financial relationships that could be construed as a potential conflict of interest.
